# Mechanically Tunable Néel Temperature in a 3D Nickel Oxide-Intercalated Muscovite Mesocrystal

**DOI:** 10.1021/acs.inorgchem.4c04498

**Published:** 2025-06-07

**Authors:** Bo-Sheng Chen, Yi-Cheng Chen, Yu-Ting Lin, Yi-Chun Chen, Cheng-En Liu, Chih-Yen Chen, Chang-Yang Kuo, Heng-Jui Liu, Tzu-Wei Wang, Po-Liang Liu, Chih-Huang Lai, Yu-Lun Chueh, Ying-Hao Chu

**Affiliations:** † Department of Materials Science and Engineering, 34881 National Tsing Hua University, Hsinchu 300044, Taiwan; ‡ Department of Chemical and Materials Engineering, National University of Kaohsiung, Kaohsiung 811726, Taiwan; § Department of Physics, 34912 National Cheng Kung University, Tainan 701401, Taiwan; ∥ Department of Electrophysics, 34914 National Yang Ming Chiao Tung University, Hsinchu 300093, Taiwan; ⊥ Department of Materials Science and Engineering, 34916 National Chung Hsing University, Hsinchu 402202, Taiwan; # Graduate Institute of Precision Engineering, National Chung Hsing University, No. 145, Xingda Road, Taichung 40227, Taiwan; ¶ Department of Applied Materials and Optoelectronic Engineering, National Chi Nan University, No. 1, University Road, Puli Township, Nantou 54561, Taiwan; ∇ College of Semiconductor Research, National Tsing Hua University, Hsinchu 300044, Taiwan; ○ Department of Materials Science and Engineering, Korea University, Seoul 02841, Republic of Korea

## Abstract

Exploring the physical properties of functional materials in response to reduced-dimensional environments has attracted significant attention. In this study, we focus on the feature of van der Waals gaps in 2D layered silicate mica for intercalation. The van der Waals gaps between mica interlayers can be considered as 2D cavities, confining the growth direction of intercalants and resulting in controlled morphology and the fabrication of well-ordered 3D mesocrystal structures of NiO. In addition to the controlled morphology, Raman spectra further reveal Néel temperature of NiO can be modulated through mechanical bending in this ambient system. Furthermore, the NiO intercalants can be reduced to Ni metal under suitable thermodynamic conditions. This research introduces a strategy to control the intercalant and paves the way for tailoring the properties of nanomaterials for specific applications, establishing the concept of flexible 3D mesocrystals.

## Graphical abstract

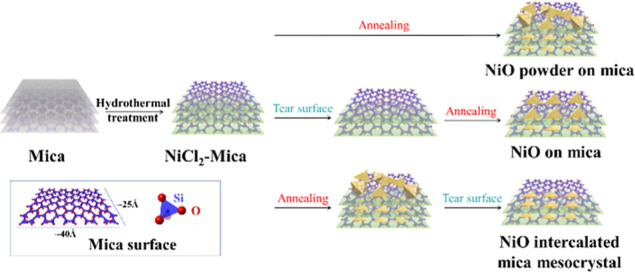

## Introduction

Muscovite, which is a layered silicate mineral, has long been utilized for its electrical and thermal insulation properties. Furthermore, its two-dimensional (2D) layered structure makes it a promising candidate for advanced electronics development and fundamental research due to its atomic flat surface and environmental stability.^1,2^ With the emergence of IoT and wearable devices in contemporary society, there has been a notable expansion in the field of mica-based flexible 3D/2D van der Waals heteroepitaxy, commonly referred to as MICAtronics.^3,4^ This field has experienced significant advancement since 2016, owing to its unique characteristics, such as high-temperature tolerance, high crystallinity, and mechanical flexibility, making it an excellent platform for the growth and integration of functional materials. The thermal stability and crystallinity of mica enable the fabrication of high-performance functional materials with a mechanical flexibility. Under the bending process, the high crystallinity ensures that defects do not absorb the applied stress but are effectively exerted on the material, allowing us to study the response of the functional thin film to external stress more effectively.

Various functional materials, such as electronics, spintronics, magnetostrictive materials, and multiferroics, have been integrated onto mica to realize flexible functional 3D/2D van der Waals heteroepitaxy with prototype demonstrations. For instance, the lead zirconium titanate/mica heterostructure demonstrates high efficiency and mechanical stability, making it suitable for flexible nonvolatile memory devices.^5^ Additionally, nonlinear dielectric materials like barium strontium titanate (BSTO) exhibit a strong coupling between the lattice structure and ferroelectric properties.^6^ Studies have demonstrated that applying strain to the BSTO lattice can modulate its charge storage capacity and ferroelectric polarization, enabling signal control through mechanical bending. This simplifies the control of the material properties and enhances device functionalities. While extensive research has focused on developing new materials for integration on the van der Waals surface of mica, less attention has been paid to mica interlayers. The van der Waals gap between the mica layers can be viewed as a 2D cavity for material intercalation. Studies have revealed that through vapor phase intercalation,^7^ insulating mica can be transformed into conductive and magnetic mica, similar to how dilute magnetic semiconductors (DMS) exhibit tunable magnetic and semiconducting degrees of freedom. Additionally, a 2D cavity can be viewed as a reduced dimension, allowing for exploration of the physical properties of intercalants in low dimensions.

Our investigation of intercalated mica can be viewed as the combination of a layered matrix and well-ordered intercalant inspired by mesocrystals. Mesocrystals, which are ordered nanocrystal superstructures, offer exciting possibilities in nanoscience and nanotechnology due to their potential applications and their tunable properties^8,9^ in catalysis, energy conversion and storage, and lithium-ion batteries. The advantage of epitaxy lies in orientation guidance and interface defect elimination, thereby preserving the properties of the material. Although mesocrystals can perform superiorly to existing materials, their single-component nature often limits them to one specific function or application. Combining two or more nanomaterials with different functions within a mesocrystal could result in multifunctional materials with synergistic properties, ideal for applications such as tandem catalysis. However, synthesizing 3D binary mesocrystals remains challenging due to the difficulties in coassembling two distinct nanocrystals^10^ arising from differences in crystallization rates. Most binary mesocrystals are formed as 2D films,^11^ often recognized as superlattices, due to limitations in assembly strategies such as solvent evaporation and thin film process. Inspired by this concept, this research proposes a new approach: achieving an ordered arrangement between guest and host materials through intercalation.

We selected nickel oxide (NiO) as the intercalant to combine with mica and explored the growth mechanisms and properties of the material. NiO is an antiferromagnetic^12^ insulator for nanoelectronic and spintronic devices.^13^ In this intercalation system, we focus on NiO’s antiferromagnetic properties, how they may change when subjected to stress and strain, and whether intercalation within the mica layers will affect its lattice. The study demonstrates the creation of NiO-intercalated mica with an epitaxial relationship, a mesocrystal-like arrangement, and related properties. Differences in NiO morphology are observed between surface-grown NiO and intercalated NiO. Analysis of thermodynamics reveals the pressure within mica interlayers and its effect on restricting intercalant growth. Temperature-dependent Raman spectroscopy confirms the antiferromagnetic nature of the NiO-intercalated mica mesocrystal and shows changes in its Néel temperature (*T*_N_). This research paves the way for epitaxial intercalation as a novel method for designing advanced 2D materials with tailored properties for various applications. It also presents an alternative approach to 3D binary mesocrystals.

## Results and Discussion

The process flow of NiO intercalation into mica involves two main steps: hydrothermal intercalation and an annealing treatment. This process is depicted in Figure 1. The hydrothermal process allows Ni ions to uniformly enter between the mica layers, while the annealing step facilitates the crystallization of NiO. In this section, we discuss the growth of NiO intercalants in different environments. NiO is a cubic structure material (rock-salt, *Fm*3̅*m*, *a* = *b* = *c* = 4.19 Å, α = β = γ = 90°). NiO(111) is the closest packed plane and exhibits the lowest surface energy in the FCC structure.^14^ According to the Wulff construction, the plane with the lowest surface energy predominantly determines the crystal morphology. Thus, the presence of the (111) plane in NiO can be predicted.

**Figure 1:**
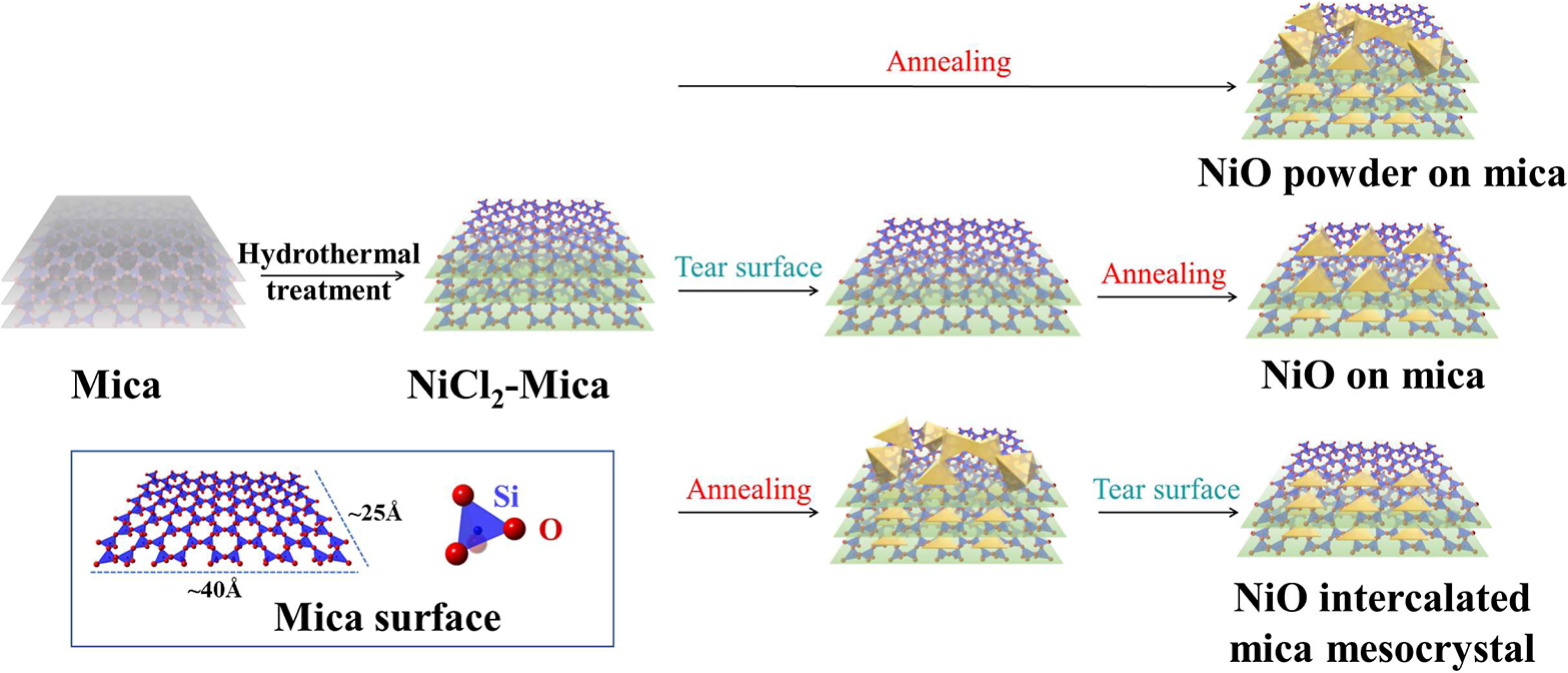
Process flow for the fabrication of different types of NiO-intercalated mica mesocrystal.

The SEM images (Figure 2A–C) demonstrate NiO crystals exhibit significantly different morphologies when grown in confined versus nonconfined environments under the same annealing process. First, octahedral NiO powders were discovered on hydrothermally intercalated mica after annealing (Figure 2A). In this case, the growth of the NiO intercalant was not restricted by the mica, allowing them to grow freely into NiO nanocrystals. Second, to further explore the effect of mica confinement, the upper surface of hydrothermal-intercalated mica was removed before annealing. Without the cover of mica, the NiO intercalant grows into a pyramid-like morphology, with some nanocrystals on top of the NiO layer retaining an octahedral shape (Figure 2B). Third, after the entire intercalation process, the top layer of mica was torn off to observe the NiO intercalant synthesized between mica layers. Due to the spatial restriction of mica, NiO grows only along the in-plane direction with a flat surface and alignment in a specific direction (Figure 2C). This ordered, flat NiO superstructure suggests the presence of intrinsic interlayer pressure that promotes the formation of flat, plate-like NiO structures, referred to as an intercalated mica mesocrystal. As a result, we focus toward exploring the relationship between Gibbs free energy and strain energy to determine the pressure associated with various basal dimensions in the NiO system. Gibbs free energy in solids primarily consists of internal and elastic energy, as the terms of pressure–volume (*PV*) and temperature–entropy (*TS*) are negligible compared to those in gases. For solids under typical conditions, the simplified Gibbs free energy is expressed as *G* = *U* + *W*_elastic_, where *W*_elastic_ represents the elastic energy from deformation. The elastic energy density is derived using Hooke’s Law, described as (1/2) *E*ε,^2^ where *E* is the elastic modulus and ε is the strain. Differentiating the Gibbs free energy with respect to strain provides the internal stress within the material. Based on this approach, we revised our original model accordingly. We employed density functional theory (DFT) calculations using the Vienna Ab initio Simulation Package (VASP) to investigate the structural stability of NiO-intercalated mica mesocrystal. The calculations utilized Generalized Gradient Approximation (GGA) with Perdew–Wang (PW91) correction.^15–18^ Optimized structural models of NiO and NiO(111) intercalation were established to evaluate energetic stability, demonstrating that combining tensile strain along the *a*- and *b*-axes with compressive strain along the *c*-axis enhances stability. This interplay of strains generates an internal tensile stress of approximately +0.0389 GPa along the *c*-axis, serving as a restoring force for structural equilibrium. The triangular plate-like morphology of NiO(111) structures arises from a balance among crystalline hexagonal symmetry, stress distribution, and energy optimization. This triangular configuration effectively disperses strain, reduces structural irregularities, and stabilizes the structure through increased restoring force. The detailed calculation is presented in the Supporting Information (Figure S1). In addition, temperature-dependent study of the morphology (Figure S2) was used to verify the formation of the NiO layer from nanoparticles.

**Figure 2:**
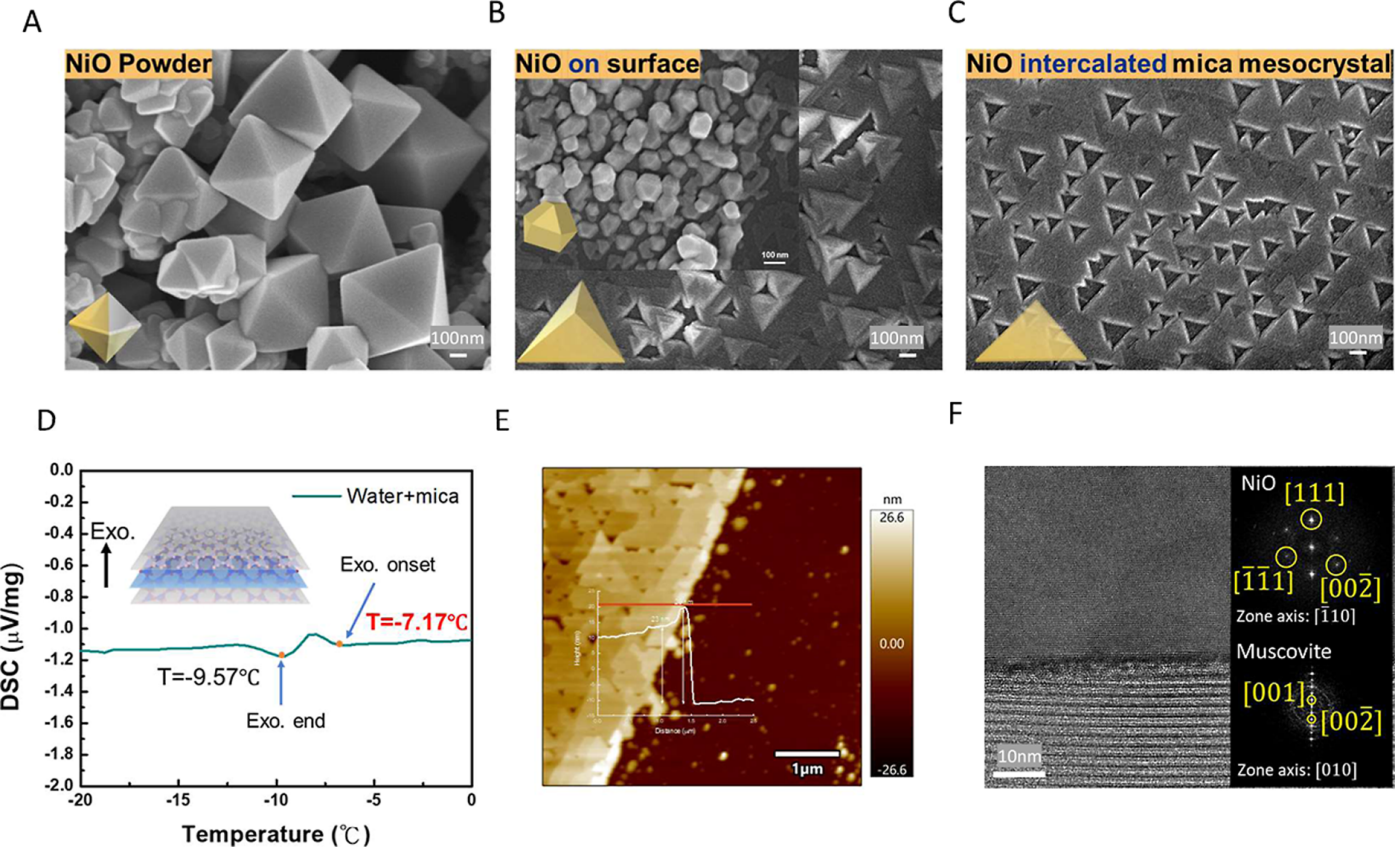
Morphology of (A) NiO powders, (B) NiO intercalant on the mica surface, and (C) NiO-intercalated mica mesocrystal. (D) The DSC measurement of water-intercalated mica. (E) AFM morphology and height. (F) The TEM images of NiO-intercalated mica mesocrystal cross-section.

To further estimate the pressure between the layers of mica, we applied water as a guest intercalant and observed its freezing point shift through differential scanning calorimetry (DSC). In Figure 2D, the DSC curve shows an exothermic reaction starting at −7.167 °C and ending at −9.567 °C. This transition represents the process of water turning into ice, with the freezing point dropping by approximately 7 °C compared to 1 atm. According to the phase diagram of water,^19^ a decrease in the freezing point by 7 °C corresponds to a pressure of approximately 0.1 GPa, which is consistent with our estimation. The results from the mathematical model and the experimental measurements are consistent, with a discrepancy of less than 1 order of magnitude. Moreover, the SEM image (Figure 2C) reveals upright and inverted triangular NiO crystals, indicating the presence of a distinct arrangement with respect to the mica layers. The triangular planar structure was further confirmed through AFM analysis. The AFM topography (Figure 2E) reveals that the surface of the intercalated NiO crystals is flat, indicating the NiO is under pressure. To further investigate the structural and compositional relationship between NiO and the mica host, TEM analysis was conducted to examine the cross-sectional structure (Figure 2F). The Fast Fourier Transform (FFT) patterns obtained from the TEM image (Figure 2F) show clear diffraction patterns, confirming the high crystallinity of the NiO intercalants. The diffraction pattern illustrates the alignment relationship between the NiO intercalants and the mica host, as follows: [2̅00]_NiO_∥[2̅02̅]_mica_, [022̅]_NiO_∥[010]_mica_, and [111]_NiO_∥[001]_mica_. The EDS mapping results, verified through SEM analysis, confirm the presence of NiO. EDS provides insight into the elemental distribution and chemical composition. As shown in Figure S3A, the elemental distribution map reveals a uniform distribution of Ni and O within the plate-like crystals. Other elements, such as Si and O, are identified as components of the mica substrate. Further investigation using TEM/EDS analysis (Figure S3B,D) shows a transit layer and weaker Ni signal near the interface. To explore this interface in more detail, a line scan was conducted (Figure S3B). The line scan result (Figure S3C) reveals a transition layer between NiO and muscovite, where signals for Mg, Al, and Si, the primary components of muscovite, are detected. This suggests potential migration or diffusion of metal cations between NiO and mica at the interface. Additionally, by comparing the X-ray absorption spectra of standard nickel oxide samples and the NiO-intercalated mica mesocrystal, it is evident that absorption peaks occur at the same energy at both the L_3_ edge and the L_2_ edge. This indicates that in our samples nickel exists as Ni^2+^, occupying octahedral sites in the crystal, consistent with the chemical coordination without the presence of Ni^3+^, as shown in Figure S3E.

To obtain the macroscopic structural information, we conducted XRD measurements further to confirm the relationship between the NiO-crystallized intercalant and mica. Through XRD theta-2theta scan (Figure 3A), only the NiO {111} series peaks can be detected at 37.2° and 79.4° in the out-of-plane direction without impure phase.^20^ Other peaks correspond to the mica {001} diffraction patterns. The rocking curve measurement characterizes the crystallinity of the NiO-crystallized intercalant. The full width at half-maximum (fwhm) of NiO (111) is 0.52° (Figure 3B). The phi scan confirms the in-plane crystallographic information. According to the results of the phi scan (Figure 3C), the diffraction peaks of NiO (200) and mica (202) are at the same position, indicating an ordered arrangement consistent with the TEM results. Additionally, NiO exhibits 3-fold symmetry. The presence of two sets of asymmetric diffraction peaks in Figure 3C is due to the formation of two differently oriented domains with NiO-crystallized intercalants arranged in opposite directions. This relationship is illustrated in Figure 3D. To further analyze the pressure applied to the NiO-crystallized intercalant, we employed reciprocal space mapping (RSM) to quantify the strain state of the NiO-crystallized intercalant. The RSM analysis revealed a compressive strain of 0.17% by comparing the NiO (111) peak to the theoretical value. This strain information was then used to calculate the pressure exerted on the NiO using the stress–strain equation: σ = *E*ε, where σ is the strain, *E* is the elastic modulus of NiO (107 GPa),^21^ and ε is the stress. Then, the calculation of strain–stress equations presents the pressure imposed on the NiO. The estimated pressure of 0.18 GPa on the NiO-crystallized intercalant is approximately 4–5 times higher than the 0.0389 GPa predicted by the Gibbs free energy model; however, both values remain within the same order of magnitude, indicating reasonable consistency between the two approaches. In addition, the RSM images displayed cyclic fringes with a periodicity of 10 nm (Figure 3E). This suggests a repeating pattern of NiO layers within the mica structure with a period of 10 nm. This value differs from the AFM data because the RSM data provide a macroscopic measurement. Since the NiO thickness exceeds the mica interlayer distance, we infer that this discrepancy may lead to the formation of stacking fault-like structures, resulting in an average periodicity of 10 nm. Analysis of the mica substrate revealed a 7% tensile strain in the out-of-plane direction (Figure 3F). The RSM analysis provided valuable (Table S1) insights into the strain–stress relationship between the NiO-crystallized intercalant and mica. The results confirm the presence of a compressed NiO-crystallized intercalant under pressure and a periodic intercalation superstructure within the host mica.

**Figure 3:**
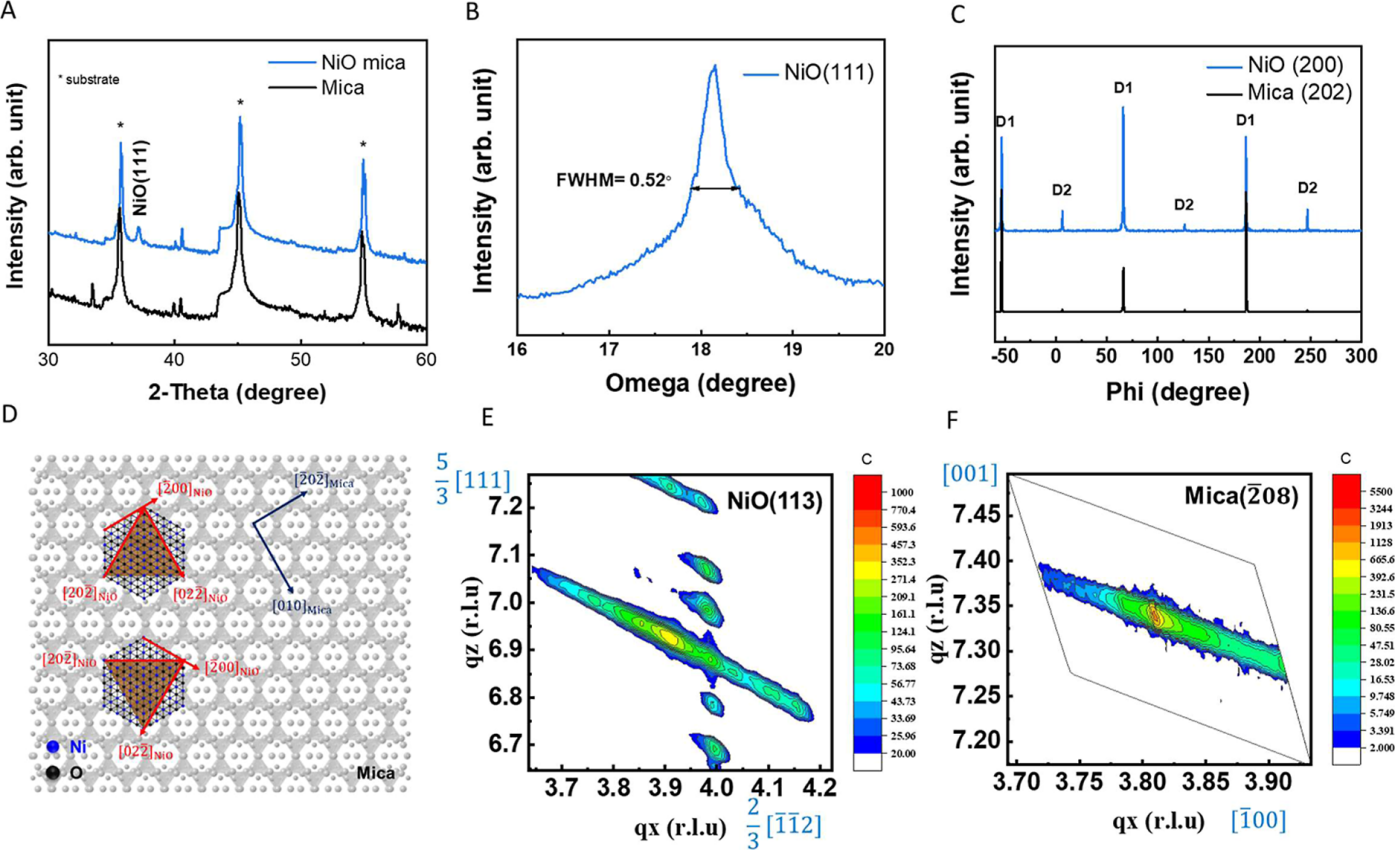
Structure analysis: (A) theta-2theta scan, (B) Rocking curve, (C) phi scan, (D) relationship of NiO and mica alignment, (E) RSM of NiO (113), and (F) RSM of mica (2̅08).

The key characteristic of the NiO-intercalated mica mesocrystal (Figure 4A) is its antiferromagnetism. Since NiO is a well-known antiferromagnetic material, it is an excellent candidate for coupling with ferromagnetic materials to form spin-valve structures for spintronic devices.^22^ Additionally, NiO is a typical p-type semiconductor suitable for applications in optoelectronic devices.^23^ However, our intercalated system is not optimized for the multilayer structures typically required for these specific applications. To explore its antiferromagnetism, we utilized synchrotron-based temperature-dependent X-ray linear dichroism (XLD). As shown in Figure 4B, the absorption intensity along the in-plane direction (*E*//*ab*) differs from that along the out-of-plane direction (*E*//*c*). This anisotropy, known as the XLD effect, confirms the presence of antiferromagnetic ordering in the NiO-intercalated mica mesocrystal. Figure S4B demonstrates a gradual decrease in the XLD at the L_2_ edge with increasing temperature. This trend is further confirmed by integrating |XLD| at the L_2_ edge, where the integral |XLD| begins to decline above 200 K. This decline suggests a weakening of the antiferromagnetic properties of the NiO-intercalated mica mesocrystal. However, additional analytical methods are required to accurately determine the *T*_N_, as NiO exhibits a relatively high *T*_N_ of 523 K. Raman spectroscopy was employed to analyze magnetic transitions, particularly in the NiO system. The vibrations of the magnons are related to the ordering of magnetic moments. Therefore, in antiferromagnetic materials, the transition from an ordered to a disordered magnetic moment can be detected by observing the appearance of the specific peak in Raman spectra.^24^ The appearance of magnons is closely related to whether a magnetic material is ordered or disordered. According to the literature, by measuring the temperature-dependent Raman spectra of NiO and observing the changes in the vibration peak positions of the two-magnon (2M) modes of nickel oxide, one can determine the Néel temperature of the sample.^25^ Figure 4D demonstrates the full range of temperature-dependent Raman spectra, measured from 300 to 475 K of the NiO-intercalated mica mesocrystal. The peaks corresponding to NiO at 503 cm^–1^ (LO), 720 cm^–1^ (2TO), 898 cm^–1^ (LO + TO), 1096 cm^–1^ (2LO), and 1468 cm^–1^ (2M) are present.^26–28^ As the temperature increases, the 2M peak broadens and moves to lower Raman shifts, while the other peaks remain unaffected by the temperature. In the NiO system, the 2M peak corresponds to spin-wave (magnon) excitations induced by antiferromagnetic interactions. This peak is directly related to the strength of the antiferromagnetic interactions. As the temperature increases, the antiferromagnetic order weakens, causing the 2M peak to decrease and eventually disappear. Once the temperature exceeds the *T*_N_, antiferromagnetic interactions disappear, and the 2M peak disappears accordingly. The *T*_N_ changes are observed in conjunction with Figures S4 and S5. Figure 4D shows that the *T*_N_ of the NiO-intercalated mica mesocrystal is 445 K. This temperature indicates the transition of NiO spin arrangement from antiferromagnetic order to paramagnetic disorder due to thermal disturbance. The measured *T*_N_ of 445 K for the NiO-intercalated mica mesocrystal falls within the expected range reported in the literature (400–490 K) for NiO nanoparticles and crystals prepared via various methods (Table S2). Additionally, we explored other potential reasons for the lower *T*_N_ compared with bulk NiO. XAS spectra confirmed that the valence state of Ni in the NiO-intercalated mica mesocrystal is 2+, with no presence of 3+ to reduce superexchange interactions, ruling out chemical coordination as the main reason. The RSM calculations show that the material experiences tensile strain, resulting in a looser atomic arrangement than the bulk material. This reduces the strength of superexchange interactions and leads to a lower *T*_N_. Consequently, after other factors were excluded, tensile stress and processing methods are identified as the primary causes for decreased *T*_N_.

**Figure 4:**
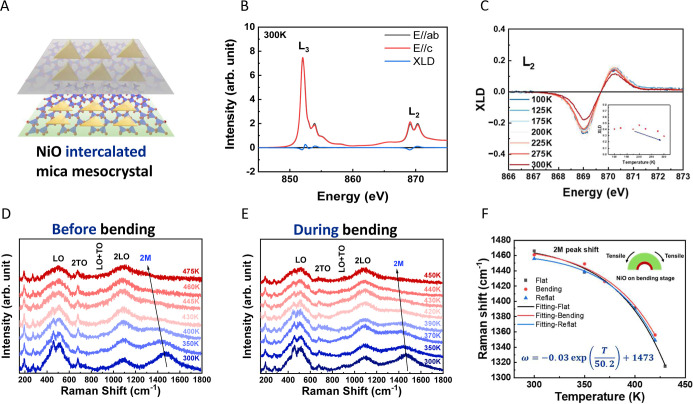
(A) Schematic of the NiO-intercalated mica mesocrystal. (B) The temperature-dependent XLD absorption spectrum. (C) Intensity change of the L_2_ edge with temperature. Temperature-dependent Raman spectra of the NiO-intercalated mica mesocrystal: (D) flat and (E) under bending. (F) Relationship between the 2M Raman shift and temperature.

Due to the mechanical flexibility of NiO-intercalated mica mesocrystal, we further investigated whether bending the sample would affect the *T*_N_. Bending induces stress that creates both compressive and tensile strain. According to the literature, when NiO is subjected to strain caused by isotropic pressure, increasing compressive pressure raises the *T*_N_ because superexchange interactions are related to the proximity of atoms.^29^ When atoms are closer, the superexchange interaction increases, raising the *T*_N_; conversely, when atoms are further apart, the superexchange interaction decreases, lowering the *T*_N_.^30^ In this experiment, a convex bending stage primarily provided tensile stress, which can be calculated using the following formula:^31^

$$\varepsilon =\left(\frac{t_{f}+t_{s}}{2R}\right)\left(\frac{1+2\eta +\chi \eta^{2}}{\left(1+\eta \right)\left(1+\chi \eta \right)}\right),,\eta =\frac{t_{f}}{t_{s}},\chi =\frac{Y_{f}}{Y_{s}}$$ 1

where ε is the strain, *t*_f_ is the thickness of NiO, *t*_s_ is the thickness of mica, *R* is the bending radius, *Y*_f_ is the Young’s modulus of NiO, and *Y*_s_ is the Young’s modulus of mica. The calculated strain is 0.0114, while the stress is 1.22 GPa. The origin *T*_N_ of the NiO-intercalated mica mesocrystal is 445 K (Figure 4D). Upon bending, *T*_N_ decreased to 430 K (Figure 4E). This indicates that the tensile stress caused by bending increases the atomic spacing, lowering the superexchange interaction and reducing the *T*_N_. When we released the sample, the *T*_N_ returned to 440 K (Figure S6), within measurement error (±3 K) of the original value, demonstrating the reversibility of the strain-induced *T*_N_ change. The relationship between Raman shift and temperature is illustrated in Figure 4F. The trend line in the graph clearly shows an exponential decrease in Raman shift with increasing temperature,^27^ confirming that temperature-induced vibrational changes influence the 2M peak in our system. From the hydrostatic pressure model, the relationship between *T*_N_ and the external pressure is as follows:

2 $$\frac{dT_{N}}{dP}=7.33\frac{K}{GPa}$$

This shows that the stress provided by the bending is around 2.05 GPa, which is on the same order of magnitude as the model estimation (1.22 GPa). The difference suggests that the stress provided by the system is not entirely isotropic. Similar to the NiO-intercalated mica mesocrystal, the 2M vibration indicates that the *T*_N_ of the NiO intercalant grown on the mica substrate surface is 440 K (Figure S7A). In contrast to the NiO-intercalated mica mesocrystal, the NiO intercalant grown on the mica shows minimal change in *T*_N_ (445 K, Figure S7B) under bending status. This indicates that the surface NiO intercalant, primarily composed of pyramid-like nanocrystals, is less affected by the strain induced by bending. After the sample was removed from the bending stage and measured again in its flat state, the 2M peak disappeared at around 440 K (Figure S7C), consistent with the pristine results. In summary, these results indicate that the antiferromagnetic properties of NiO can be modulated and reversible through bending. The *T*_N_ of the NiO-intercalated mica mesocrystal decreases under bending, weakening the superexchange interaction, whereas the *T*_N_ of the NiO intercalant grown on the mica substrate remains unaffected by stress. The temperature-dependent Raman spectroscopy measurements of *T*_N_ are summarized in Table 1. Although previous studies have reported that mechanical stress can influence physical properties,^32^ bulk NiO crystals are not flexible; therefore, only hydrostatic pressure can be applied, limiting their practical applications. In contrast, the intercalation of NiO mesocrystal provides a viable approach to controlling crystal properties. Although the observed change in *T*_N_ is modest, it demonstrates that the intercalated system offers the potential platform for studying strain-sensitive materials under pressure.

**Table 1:** Summary of *T*_N_ Change under Different Bending Conditions

sample	before bending (K)	during bending (K)	after bending (K)
NiO-intercalated mica mesocrystal	445	430	440
NiO on mica	440	445	440

After characterization, we aimed to explore whether the properties of the 3D mesocrystal could be tuned under confined environments. For the NiO-intercalated mica, annealing the sample in a reducing atmosphere converts NiO into metallic Ni, resulting in the fabrication of Ni-intercalated mica. XRD confirms the crystalline characteristics of the metallic nickel. The theta-2theta scan in Figure S8A shows an out-of-plane Ni(111) diffraction peak at 44°, with no other crystal planes detected, confirming the successful reduction of NiO to metallic nickel while maintaining single-crystal characteristics. The in-plane phi scan shows that the Ni(200) and mica (202) diffraction peaks are aligned, indicating an epitaxial relationship between the metallic nickel and mica, as shown in Figure S8B and Figure S8C. According to the results of XRD, we can obtain the relationship between Ni and mica: [2̅00]_Ni_∥[2̅02̅]_mica_, [022̅]_Ni_∥[010_mica_, and [111]_NiO_∥[001]_mica_. This alignment matches the TEM diffraction pattern (Figure S8D). EDS showed minimal oxygen, confirming complete reduction, while AFM revealed spherical Ni nanocrystals (Figure S8F). SEM (Figure S9A) and EDS (Figure S9B,C) further confirmed Ni, with Si from the mica substrate. XMCD analysis (Figure S10) confirmed the ferromagnetism of the sample, with Ni contributing to the magnetism. VSM was used to study the macroscopic magnetic properties and strain response of Ni-intercalated mica. In-plane and out-of-plane (IP and OOP) M-H loops (Figure S11A,B) confirmed ferromagnetism, with no change in saturation magnetization under different bending radii (Figure S11C,D). Even after 500 bending cycles, the saturation magnetization remained stable (Figure S12), indicating high magnetic stability against external strain. Electrical transport measurements confirmed the metallic conductivity of Ni-intercalated mica, with resistance decreasing monotonically as the temperature decreased (Figure S13). This demonstrates that such guest intercalants can be used to obtain materials with different valence states by controlling the annealing environment. The physical properties of the NiO-intercalated muscovite system can be modified from antiferromagnetism to ferromagnetism through proper thermal treatment. Similar to how thermal treatment can modify the conductivity of substrates, this tunability enhances the potential functionalities and applications when integrating mesocrystal into devices.

## Conclusion

In this study, we developed an intercalation chemistry method to synthesize NiO-intercalated mica mesocrystal, enabling a detailed investigation of their morphology, structure, and material properties. The unique van der Waals gaps in 2D layered mica act as confined cavities that influence the growth of NiO into a flat triangular morphology. This internal pressure, confirmed by RSM and DSC measurements, facilitates the formation of a well-ordered 3D mesocrystals structure, and XLD spectroscopy verifies its antiferromagnetic nature. Temperature-dependent Raman spectroscopy established a *T*_N_ of approximately 445 K, which can be tuned to 430 K through mechanical bending. Although the modulation is not large, it highlights the potential of our ambient-stable pressure platform to induce controllable strain and enables seamless integration into measurement systems without requiring the design of a new cell or the use of additional pressurizing medium to maintain the desired conditions. Additionally, under a reducing atmosphere, the NiO intercalants can be converted to Ni, resulting in the emergence of ferromagnetic and metallic conductivity. Our findings demonstrate that intercalation within mica not only facilitates the growth of ordered 3D NiO mesocrystal but also enables strain modulation. This approach offers a promising route for fabricating highly ordered crystalline materials with tailored physical characteristics, integrating material growth control and strain modulation and paving the way for further exploration of flexible 3D mesocrystals in advanced functional applications.

## Methods

### Sample Fabrication

We cut the 180 μm thick, 5 cm × 5 cm fluorophlogopite mica into approximately 3 mm × 4 mm pieces using scissors. After the excess mica powder was blown off with an air blower, the pieces were placed into a Teflon-lined autoclave to facilitate the subsequent hydrothermal process. In the meantime, we dissolved NiCl_2_·6H_2_O (96.0%, Showa Chemical Industry Co., Ltd.) in deionized water to prepare a 3 M, 50 mL green solution. NiCl_2_·6H_2_O is highly soluble; it allows for precise control of the precursor concentration and a high degree of dissociation in solution. We placed the solution and the cut mica pieces into a 100 mL Teflon liner and then sealed it within a stainless steel autoclave. The autoclave was heated in an oven at a rate of 2.5 °C/min to 100 °C, where a hydrothermal reaction was maintained for 24 h. After the reaction, the system was cooled to room temperature at a rate of 2.5 °C/min to complete the diffusion of Ni ions into the mica.

After the hydrothermal treatment, the samples were placed between two glass slides and dried slowly in an oven at 80 °C to remove the water. The dried sample was then placed in an alumina crucible and subjected to thermal treatment in the furnace. The thermal treatment involved heating at a rate of 5 °C/min to 900 °C in an atmospheric environment, holding this temperature for 8 h to allow for oxidation, and cooling to room temperature at a rate of 5 °C/min to obtain NiO-intercalated mica. To further process the NiO-intercalated mica into metallic Ni-intercalated mica, the samples were placed in an alumina crucible and transferred to a tube furnace. The system was evacuated to a vacuum level of 5 × 10^–3^ Torr, and a 5% H_2_–Ar gas mixture was introduced at a flow rate of 0.5 sccm. The samples were heated at a rate of 5 °C/min to 900 °C, held at this temperature for 8 h for the reduction reaction, and then cooled to room temperature at a rate of 5 °C/min, yielding metallic nickel-intercalated mica.

### Structural Analysis

Synchrotron-based X-ray diffraction (beamline 17B) confirmed the crystal structure and epitaxial relationships at the National Synchrotron Radiation Research Center, Taiwan. The focused ion beam technique prepared and examined the TEM specimen using a JEOL JEM-F200 microscope. The JSM-IT800 instrument collected the SEM and EDS data in the Center for Nanotechnology, Materials Science and Microsystems, NTHU.

### Thermal Analysis

The freezing point of water-intercalated mica was measured by simultaneous thermogravimetry and differential scanning calorimetry (STA PT 1750C, Linseis).

### Magnetic Measurements

XAS, XMCD, and XLD experiments were performed at the NSRRC-MPI TPS 45A Submicrometer Soft X-ray Spectroscopy beamline in Taiwan. The IP and the OOP macroscopic magnetic hysteresis loops were characterized by VSM.

### Transport Measurements

Temperature-dependent resistance measurements were performed using a PPMS by Quantum Design based on a standard four-probe technique.

### Bending Tests

Temperature-dependent Raman measurements were conducted using a backscattering confocal microscope combined with a spectrometer (iHR550, Horiba Jobin Yvon), and the excited source was a 532 nm solid-state laser. A long working distance 50× objective lens (NA = 0.5) was used in the temperature-dependent Raman measurements, with the sample fixed on homemade molds with various bending radii. A Gaussian fitting method was employed to analyze the Raman peaks.

### Theoretical Modeling

The stability of the NiO-intercalated mica mesocrystal was investigated by using first-principles density functional theory (DFT) calculations implemented in the Vienna Ab initio Simulation Package (VASP). The Generalized Gradient Approximation (GGA) described the exchange–correlation interactions with the Perdew–Wang (PW91) correction.

## Supplementary Material



## Notes

Additional calculation details, analysis procedures, composition characterization, comparison tables, experimental details, and materials and methods (PDF)
