# Supplementary material for: Mechanically Tunable Néel Temperature in a 3D Nickel Oxide-Intercalated Muscovite Mesocrystal
Source: Inorg Chem. 2025 Jun 7;64(24):11898–906. doi: 10.1021/acs.inorgchem.4c04498 (PMC12188562; doi:10.1021/acs.inorgchem.4c04498)
Supplement: Supplementary file 1 [file ic4c04498_si_001.pdf]

Supporting Information

Mechanically Tunable Néel Temperature in a 3D Nickel Oxide-Intercalated Muscovite Mesocrystal

Bo-Sheng Chen[†], *Yi-Cheng Chen*[*,†,‡], *Yu-Ting Lin*[§], *Yi-Chun Chen*[§], *Cheng-En Liu*[∥], *Chih-Yen Chen*[∥], *Chang-Yang Kuo*[∥], *Heng-Jui Liu*[⊥], *Tzu-Wei Wang*[#], *Po-Liang Liu*[#,¶] *Chih-Huang Lai*[†], *Yu-Lun Chueh*[†,▽,○] and *Ying-Hao Chu*[*,†,▽]

[†]Department of Materials Science and Engineering, National Tsing Hua University, Hsinchu, 300044, Taiwan

[‡]Department of Chemical and Materials Engineering, National University of Kaohsiung, Kaohsiung, 811726, Taiwan

[§]Department of Physics, National Cheng Kung University, Tainan, 701401, Taiwan

[∥]Department of Electrophysics, National Yang Ming Chiao Tung University, Hsinchu, 300093, Taiwan

[⊥] Department of Materials Science and Engineering, National Chung Hsing University, Hsinchu, 402202, Taiwan

[#]Graduate Institute of Precision Engineering, National Chung Hsing University, No. 145, Xingda Road, Taichung, 40227, Taiwan.

[¶]Department of Applied Materials and Optoelectronic Engineering, National Chi Nan University, No. 1, University Road, Puli Township, Nantou, 54561, Taiwan

[∇]College of Semiconductor Research, National Tsing Hua University, Hsinchu, 300044, Taiwan

[○]Department of Materials Science and Engineering, Korea University, Seoul, 02841, Republic of Korea

KEYWORDS: NiO, intercalation, mesocrystal, antiferromagnetic, mica

*Email: ycchen@nuk.edu.tw. and yhchu@mx.nthu.edu.tw.

We utilize the relationship between Gibbs free energy and strain energy to determine the pressure corresponding to different basal dimensions in the NiO system. The basic definition of Gibbs free energy is G = U + PV – TS, where U, PV, and TS are the internal energy, the pressure-volume work, and the product of temperature and entropy, respectively. In solids, the effects of the PV and TS terms on Gibbs free energy are much smaller than those in gases due to the inherent properties of solids. For gases, the PV term is significant because gases are highly compressible, and their volume changes substantially with pressure according to the ideal gas law PV=nRT, where n and R are the number of moles of the gas and the ideal gas constant, respectively. In contrast, solids have extremely low compressibility, characterized by a high bulk modulus, meaning their volume remains nearly constant even under considerable pressure. As a result, the PV term in solids is negligible under normal conditions, contributing almost nothing to the Gibbs free energy. Only under extremely high pressures, such as several GPa or higher, does this term become relevant. The TS term also exhibits a substantial difference between gases and solids. For gases, entropy S is large due to the high degree of molecular freedom and the vast, accessible volume, resulting in a considerable TS contribution to the Gibbs free energy, especially at high temperatures. This term significantly influences the Gibbs free energy in gases. However, in solids, entropy mainly arises from the vibrational entropy of atoms, which is much smaller in magnitude. The TS term for solids becomes noticeable only at very high temperatures or during phase transitions, such as melting or sublimation. At typical temperatures, the entropy contribution remains relatively small. Consequently, the Gibbs free energy of solids under standard conditions is effectively dominated by the internal energy U and elastic energy contributions W_{elastic}. The simplified expression for the Gibbs free energy of a solid is G = U + W_{elastic}. The elastic energy of a solid

arises from the elastic deformation of internal atomic bonds when the material is subjected to stress. This stored energy can be derived from Hooke's Law and the stress-strain relationship. In the elastic deformation regime, stress and strain are related by Hooke's Law, which states $\sigma = E\varepsilon$, where E is the elastic modulus, which measures the material's stiffness, also measured in Pa (N/m^2). In this expression, σ is the stress (measured in Pa or N/m^2), ε is the strain (a dimensionless quantity) defined as the relative deformation $\varepsilon = \Delta L / L$, where L is the original length, and ΔL is the change in length. The strain energy density, which is the elastic energy stored per unit volume, can be defined as the integral of stress with respect to strain from zero to the applied strain: $u = \int_0^\varepsilon \sigma \; d\varepsilon$. Using Hooke's Law, this becomes $u = \int_0^\varepsilon E\varepsilon \; d\varepsilon. = (1/2)\, E\, \varepsilon^2$. This result shows that, within the elastic regime, the strain energy density is proportional to the square of the strain and the material's stiffness as quantified by Young's Modulus. It also indicates that the stored elastic energy increases quadratically with strain, meaning that even small increases in strain can lead to significant increases in stored energy. Therefore, the elastic energy contribution W_{elastic} can be expressed as the product of the energy density and the strained volume of the material or can be written as $W_{\text{elastic}} = u\text{V} = (1/2)\ E\ \varepsilon^2\text{V}$. Consequently, the Gibbs free energy of solids under standard conditions is $\text{G} = \text{U} + (1/2)\, E\, \varepsilon^2\text{V}$. To find how the Gibbs free energy changes with strain, we need to differentiate the Gibbs free energy G with respect to strain ε. This differentiation aims to determine the internal stress σ generated within the solid as a result of the applied strain. This approach is based on the thermodynamic definition of stress, where stress is the rate of change of free energy with respect to strain. Taking the derivative of G with respect to ε: $\text{dG}/\text{d}\varepsilon = E\varepsilon\text{V} = \sigma\text{V}$. Therefore, the stress is $\sigma = (1/\text{V})\ (\text{dG}/\text{d}\varepsilon)$. This indicates that the rate of change of Gibbs free energy with respect to strain is exactly the stress.

The stability of the NiO intercalated mica mesocrystal was thoroughly investigated using first-principles density functional theory (DFT) calculations implemented in the Vienna *Ab initio* Simulation Package (VASP). The Generalized Gradient Approximation (GGA) described the exchange-correlation interactions with the Perdew-Wang (PW91) correction[1-4]. This study focused on comprehensive structural optimization and energetic stability assessment to evaluate the impact of NiO intercalation on the stability of the mica mesocrystal. The DFT calculations provided critical insights into the structural modifications and energy changes associated with the intercalation process, enabling a deeper understanding of the interaction mechanisms and potential applications of the NiO intercalated mica mesocrystal. The NiO unit cell model consists of 8 atoms, including 4 Ni and 4 O atoms, with a space group of $Fm\bar{3}m$. The plane-wave cut-off energy was set to 450 eV, and the Brillouin zone was sampled using a 13 × 13 × 13 Monkhorst-Pack grid to ensure sufficient calculation accuracy. Subsequently, the optimized NiO structure model was utilized to construct a NiO(111) intercalation model. The NiO(111) intercalation model comprises 24 atoms, where 12 atoms are Ni and 12 are O. To achieve reliable intercalation energy calculations, a plane-wave cut-off energy of 450 eV was employed, and the Brillouin zone was sampled using a 5 × 5 × 3 Monkhorst-Pack grid. The electronic structure calculations were performed considering the valence electron configurations of Ni ($3d^8\ 4s^2$) and O ($2s^2 2p^4$). The optimization process aimed to obtain an energetically stable intercalation structure that accurately reflects the physical and chemical characteristics of the NiO(111) intercalation. This model will serve as the basis for further investigations of the intercalation process, particularly focusing on the influence of intercalation-induced stress on the Gibbs free energy of the NiO(111) system. The optimized model will be fundamental for evaluating how structural deformation and

stress from intercalation affect the thermodynamic stability of the NiO(111) intercalation.

Figure S1 illustrates the free energy variation and structural stability of NiO(111) under different strain conditions on a Mica substrate. The analysis focuses on the strain-induced changes in both in-plane (*a*, *b* axes) and out-of-plane (*c* axis) directions, highlighting the stability of the NiO(111) structure under various strain states. The figure consists of two primary energy-strain curves and two structural models. The lower energy-strain curve describes the variation of Gibbs free energy along the *a*- and *b*-axes, leading to a more stable NiO(111). The horizontal axis represents the applied strain (ε) in the *a* and *b* directions, ranging from −0.3 to 0.3, where negative values indicate compressive strain and positive values indicate tensile strain. The vertical axis shows the free energy G in eV. The black dashed curve, fitted by a quadratic polynomial, is described by the equation: $G = 0.0255\varepsilon^2 - 0.0069\varepsilon - 137.47$. This curve exhibits a typical parabolic shape, indicating a nonlinear variation of free energy with respect to the applied strain. The lowest point of the curve corresponds to the bottom right structural model or the most stable state of the NiO(111) structure under free strain conditions. The figure's blue arrow points towards the lowest point of the curve in the lower plot when NiO(111) is subjected to 1% tensile strain along the *a*- and *b*-axes, with further strain optimization along the *c*-axis, corresponding to the optimized structure shown at the top left. The upper right energy-strain curve illustrates the Gibbs free energy variation of NiO(111) under different out-of-plane (*c*-axis) strain conditions. The fitting curve indicates that compressive strain along the *c*-axis results in the lowest Gibbs free energy, which is expressed by $G = 0.0168\varepsilon^2 + 0.0523\varepsilon - 137.38$. The results indicate that combining tensile strain along the *a*- and *b*-axes with compressive strain along the *c*-axis leads to enhanced stability of the

NiO(111) structure. This improved stability arises from the restoring force generated by the *c*-axis tensile stress, which counterbalances the in-plane tensile strain through an energy minimization process. The energy-strain curves exhibit a parabolic shape, where the minimum energy point corresponds to the most stable configuration of the NiO(111) structure. This parabolic behavior reflects the structure's inherent elastic response, where the stored strain energy is effectively minimized under specific strain conditions. Notably, when the NiO(111) surface experiences approximately 1% tensile strain along the *a* and *b* directions, the structural stability along the *c*-axis is significantly influenced by the induced restoring force acting to maintain equilibrium. The structural models presented in the figure further illustrate how the interplay between in-plane tensile strain and out-of-plane compressive strain contributes to the overall stability of the NiO(111) intercalation. These models demonstrate how the restoring force along the *c*-axis aids in redistributing strain energy, thereby promoting the formation of triangular NiO structures with optimized stability. To determine the internal stress σ associated with the applied strain, we apply the thermodynamic definition of stress σ = (1/V) (dG/dε). Calculating the derivative of G with respect to ε: dG/dε = 2(0.0168)ε + 0.0523. When evaluated at the given out-of-plane strain ε = −0.0017, the stress is: dG/dε = 2×(0.01168)(−0.0017) + 0.0523 = +0.052243 eV/Å^3. Converting this value to GPa using the conversion factor (1 eV/Å^3 ≈ 160.2 GPa): σ = [(1/214.914) × 0.052243] × 160.2 ≈ +0.0389 GPa. This calculation shows that the internal stress σ is approximately +0.0389 GPa, indicating tensile stress within the NiO(111) structure along the *c*-axis. When the NiO(111) structure is subjected to tensile strain along the *a* and *b* axes (approximately 1%), the system attempts to preserve its overall structural stability. According to Poisson’s effect, this in-plane tensile strain induces a compressive strain along the *c*-axis. This compression results from the natural tendency

of materials to conserve volume or minimize volume change when deformed. The internal stress along the c-axis, calculated to be +0.0389 GPa, represents a restoring force that the material generates to counteract the applied compressive strain. This force is not an externally applied stress but rather an intrinsic elastic stress arising from the material's attempt to restore its original equilibrium state. The presence of the restoring force plays a critical role in supporting the formation of the triangular-shaped NiO structures. When the triangular plate-like NiO structure forms, this particular morphology provides a larger triangular area to distribute the in-plane tensile strain effectively. As the growth process continues, if the in-plane expansion of the triangular structure is suppressed, increasing the triangular area can enhance the restoring force. This enhanced restoring force, which acts along the c-axis, stabilizes the structure by counterbalancing the in-plane strain-induced compression. The mechanism behind this phenomenon is that the restoring force is directly related to the strain energy stored within the structure. A larger triangular area allows the system better to distribute the strain energy across a wider surface, thereby increasing the magnitude of the restoring force acting to pull the c-axis back to its equilibrium length. The formation of triangular-shaped NiO(111) structures can be attributed to the intrinsic characteristics of the crystal structure and its energy optimization mechanism. NiO(111) corresponds to a specific crystal plane of the face-centered cubic (FCC) structure. In this crystalline arrangement, the (111) plane exhibits hexagonal symmetry, strongly influencing the intercalated structures' morphology. The triangular shape is a natural manifestation of hexagonal symmetry because a triangle represents the simplest geometric unit compatible with hexagonal symmetry. When triangular structures grow, they align with the underlying NiO(111) lattice, thereby minimizing the surface energy. This symmetry-matching process significantly reduces the formation of high-energy edges

or irregular boundaries, which would otherwise occur if the structures were to form as squares or circles. Furthermore, the formation of triangular structures is also driven by the dynamic balance between growth rate and internal stress. As the NiO(111) structures grow within the confined environment of the mica substrate, the triangular shape allows for efficient strain distribution across the three edges. The freedom to adjust each edge's length effectively disperses the in-plane tensile strain, unlike square or circular shapes, which would result in uneven stress distribution and higher energy states. From a thermodynamic perspective, the triangular shape is the most favorable configuration for achieving energy minimization. The triangular structures enhance the restoring force that balances the internal strain along the *c*-axis by providing a larger surface area that can be expanded during growth. This synergistic interaction between restoring force, strain distribution, and crystalline symmetry results in stable triangular-shaped NiO(111) structures. The triangular plate-like structures actively adjust their configuration to achieve stability by redistributing internal stress toward a more energetically favorable configuration. The confined environment between mica layers promotes the formation and stability of the triangular morphology by providing a controlled setting that limits structural distortions. As intercalation progresses, the system continually adjusts to maintain an optimal energy state through the balance of strain and stress, offering valuable insights into optimizing intercalation processes for improved material performance.

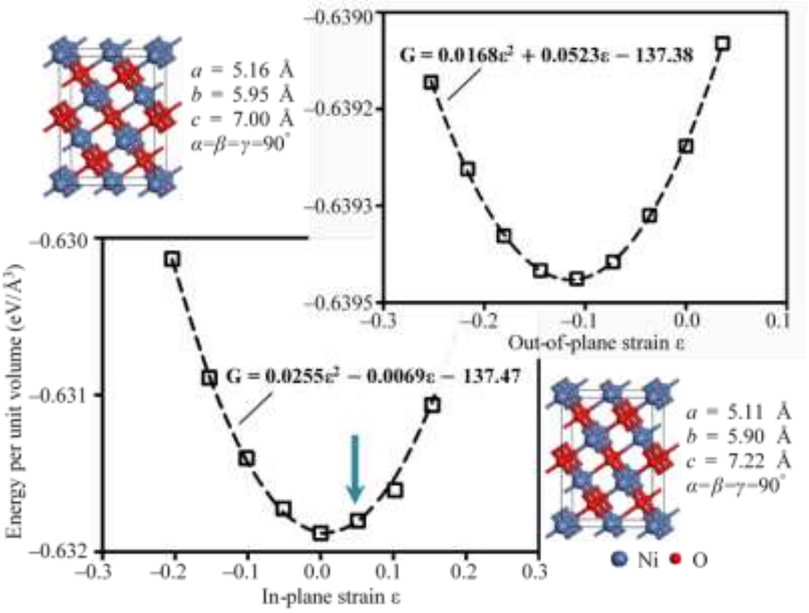

Figure S1. Gibbs free energy variation and structural stability of NiO(111) on a mica substrate under different strain conditions. Both curves represent the strain-energy relationship described by fitting equations with determination coefficients of R^2 =0.99. The curves illustrate how Gibbs free energy varies with out-of-plane (*c*-axis) strain when NiO(111) is subjected to different in-plane tensile strain conditions along the *a*- and *b*-axes. The structural models indicate the atomic arrangements corresponding to the energy minima under specific strain conditions.

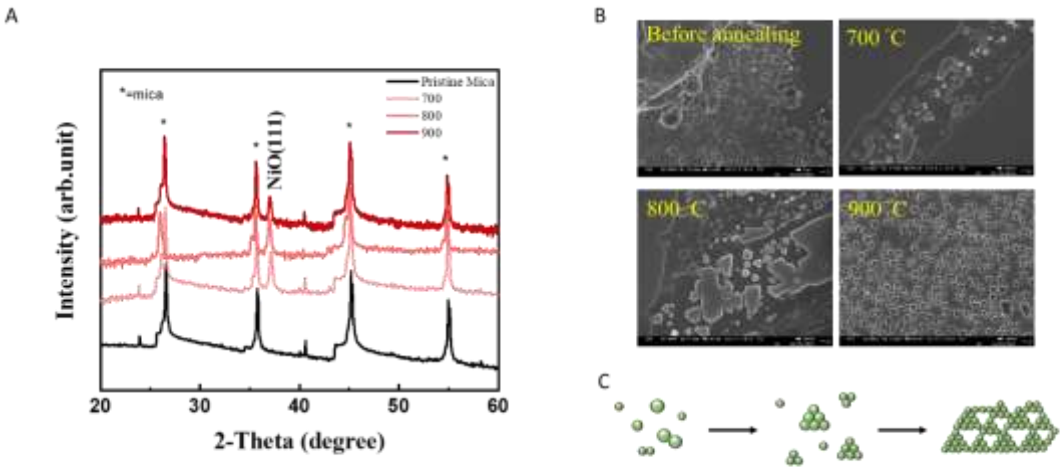

Figure S2. (A) Temperature-Dependent XRD. (B) SEM morphology at different temperatures. (C) Schematic diagram of the growth process

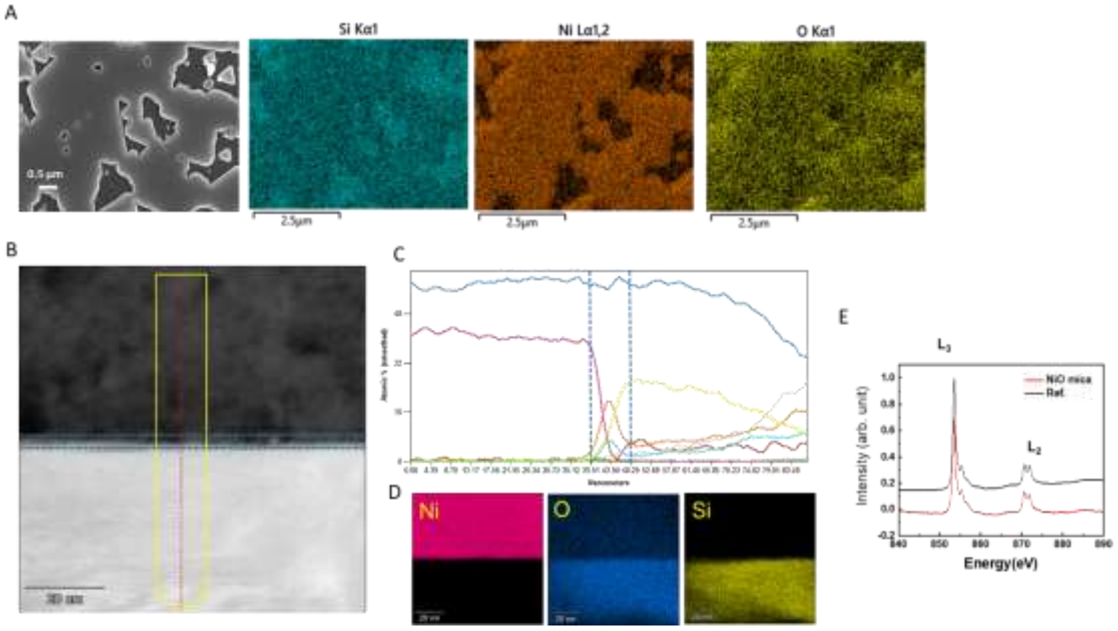

Figure S3. Composition of NiO intercalated mica mesocrystal: (A)The SEM/EDS mapping, (B) The cross-section TEM image, (C) The line-scan EDS spectra, (D)The TEM/EDS mapping, (E) XAS of the Ni $L_{2,3}$ edge.

Table S1. The analysis of strain exerted on NiO using the RSM approach.

Material	OOP	OOP%	IP	IP%
NiO bulk	$d_{NiO(111)} = 2.404Å$		$d_{NiO(11\bar{2})} = 1.700Å$	
NiO intercalated mica	$d_{NiO(111)} = 2.4Å$	0.166%(compress)	$d_{NiO(11\bar{2})} = 1.708Å$	0.47% (tensile)
Mica	$d_{Mica(001)} = 10.88Å$	7.3%(tensile)	$d_{Mica(100)} = 5.24Å$	0.19% (compress)

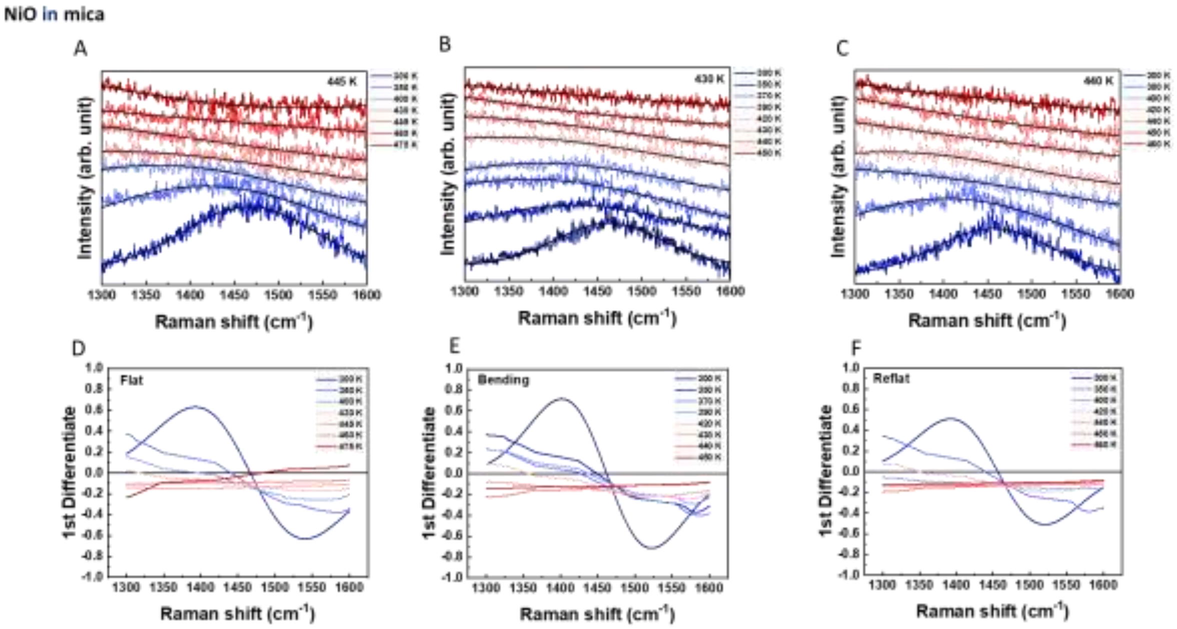

Figure S4. Temperature-dependent Raman spectra of the 2M peak of NiO-intercalated mica mesocrystals under different bending states: (A) flat, (B) bending, and (C) reflat. The 1st-order derivative was used to identify the transition points for each state: (D) flat, (E) bending, and (F) reflat.

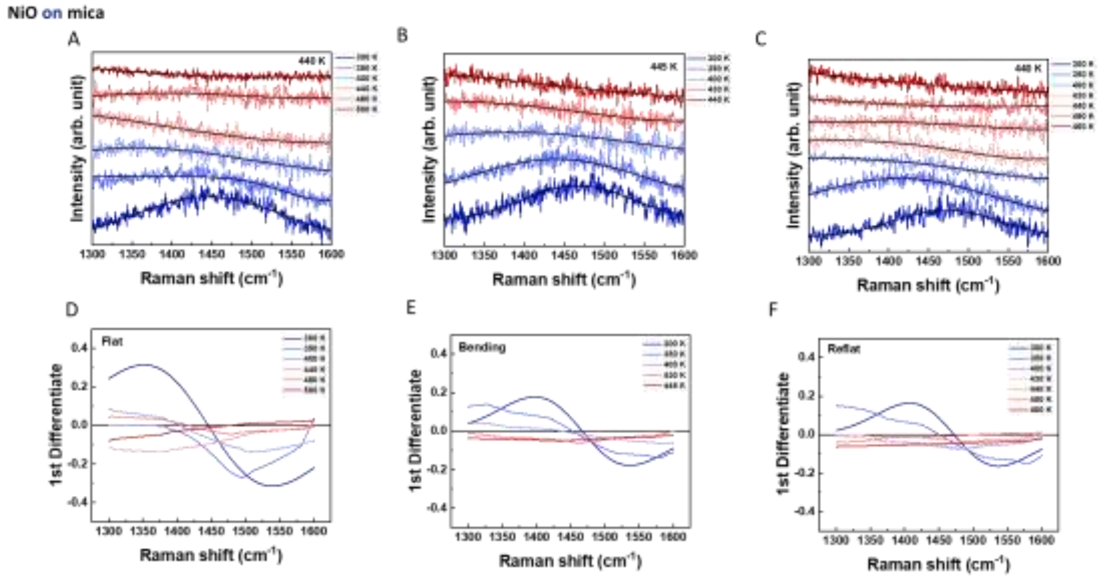

Figure S5. Temperature-dependent Raman spectra of the 2M peak of NiO on mica under different bending states: (A) flat, (B) bending, and (C) reflat. The 1st-order

derivative was used to identify the transition points for each state: (D) flat, (E) bending, and (F) reflat.

Table S2. Comparison of T_N for synthesized NiO nanocrystals

**Sample**	**Precursor**	**Method**	**Particle size (nm)**	T_N **(K)**	**Ref.**
Bulk NiO				523	[5]
NiO NPs	$Ni(NO_3)_2{\cdot}6H_2O$	Thermal decomposition	72	480-530	[6]
NiO NPs in silica matrix	$Ni(NO_3)_2{\cdot}6H_2O$	Sol-gel	5	56	[5]
NiO NPs	$Ni(NO_3)_2{\cdot}6H_2O$	Hydrothermal	6.8	488	[7]
NiO NPs	$Ni(OH)_2$	Solution precipitation calcining	43.5	492	[8]
NiO NPs	$Ni(OH)_2$	Solution precipitation calcining	5.7	435	[8]

NiO nano-disc	$Ni(OH)_2$	Solution precipitation calcining	12	460	[9]
NiO NPs	Ni powders	Calcining	20-60	480	[10]
NiO NPs	$Ni(NO_3)_2 \cdot 6H_2O$	Solution precipitation calcining	16-25	30	[11]
NiO NPs	Ni_2SO_4	Co-precipitation calcining	1-2	35	[12]
NiO NPs	$Ni(OH)_2$	Solution precipitation calcining	6	300	[13]
NiO NPs	$Ni(NO_3)_2 \cdot 6H_2O$	Hydrothermal	7	400	[14]
NiO intercalated mica	$NiCl_2 \cdot 6H_2O$	Hydrothermal annealing	20	445	This work

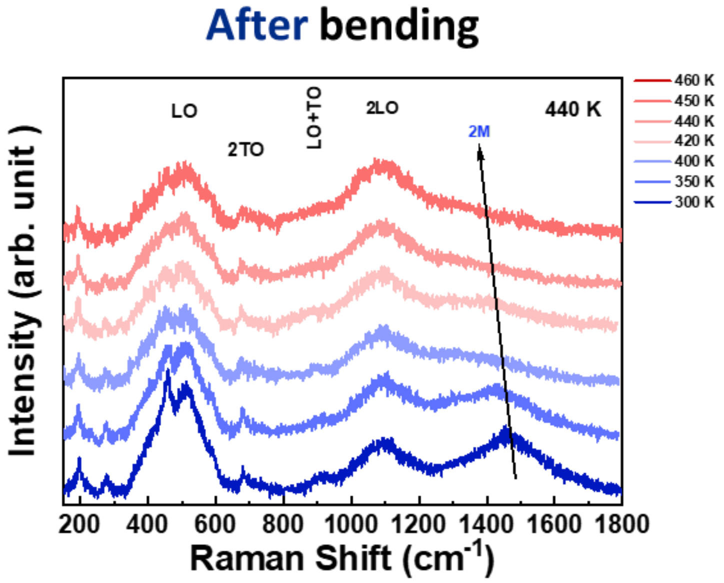

Figure S6. Temperature-dependent Raman spectra of NiO intercalated mica mesocrystal: release

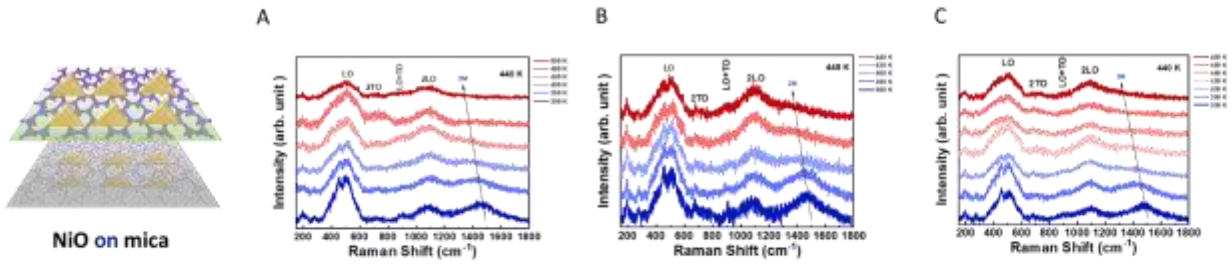

Figure S7. Temperature-dependent Raman spectra of NiO intercalant grown on mica surface: (A) flat, (B) under bending, (C) release

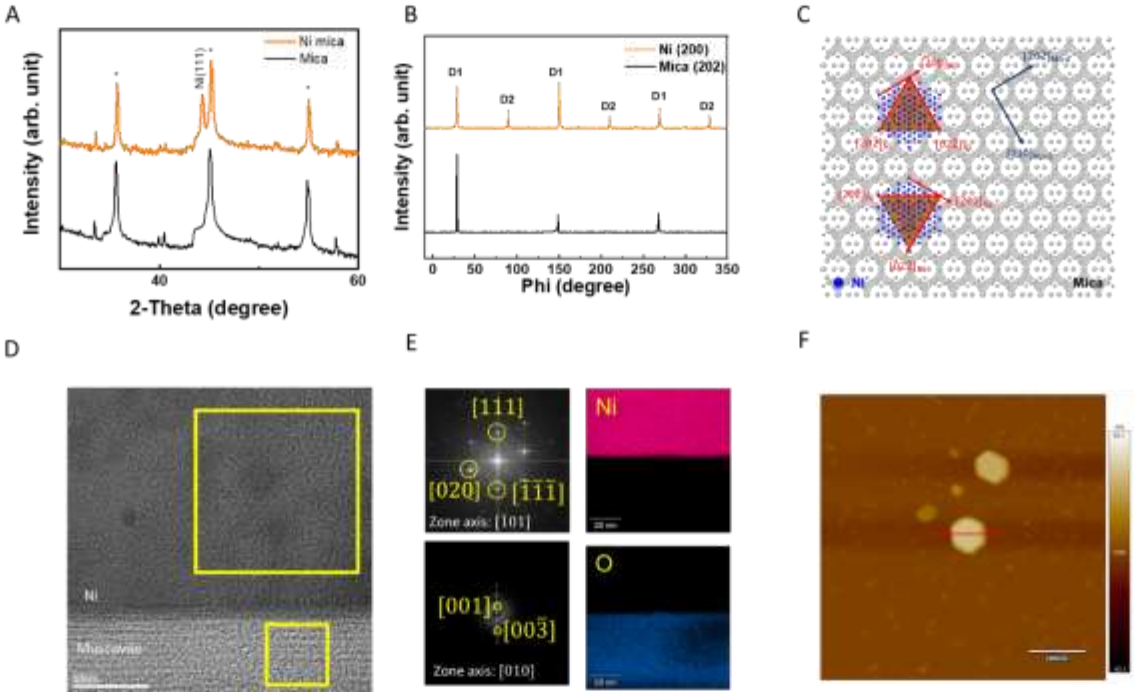

Figure S8 Structural information of Ni intercalated mica: (A) theta-2theta scan, (B) phi scan, (C) schematic of crystallographic, (D) the TEM images of Ni nanocrystal intercalated mica cross-section and (E) the corresponding FFT patterns and EDS mapping, (F) AFM morphology.

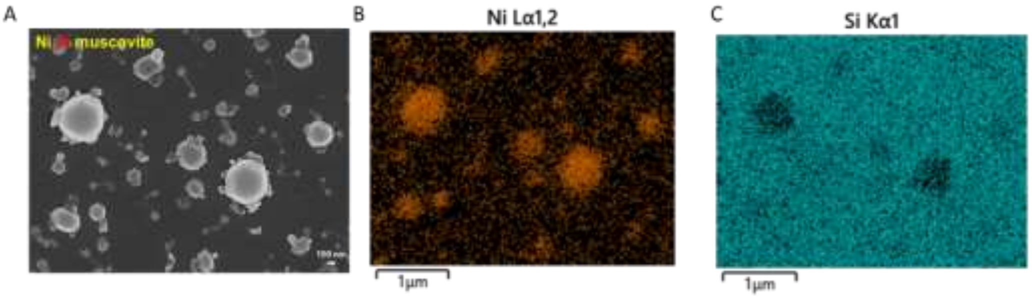

Figure S9. The Ni nanocrystal intercalated mica: (A) SEM morphology, elemental mapping of (B) Ni and (C) Si.

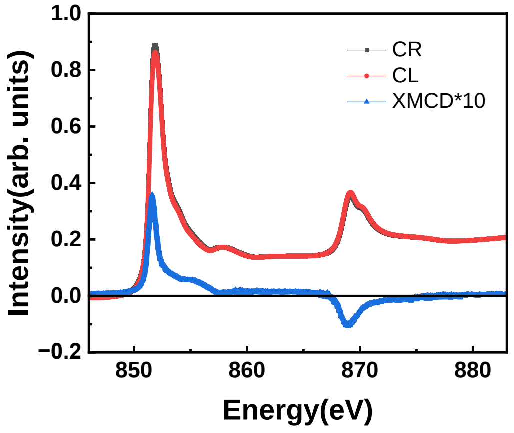

Figure S10. XMCD spectra of Ni intercalated mica

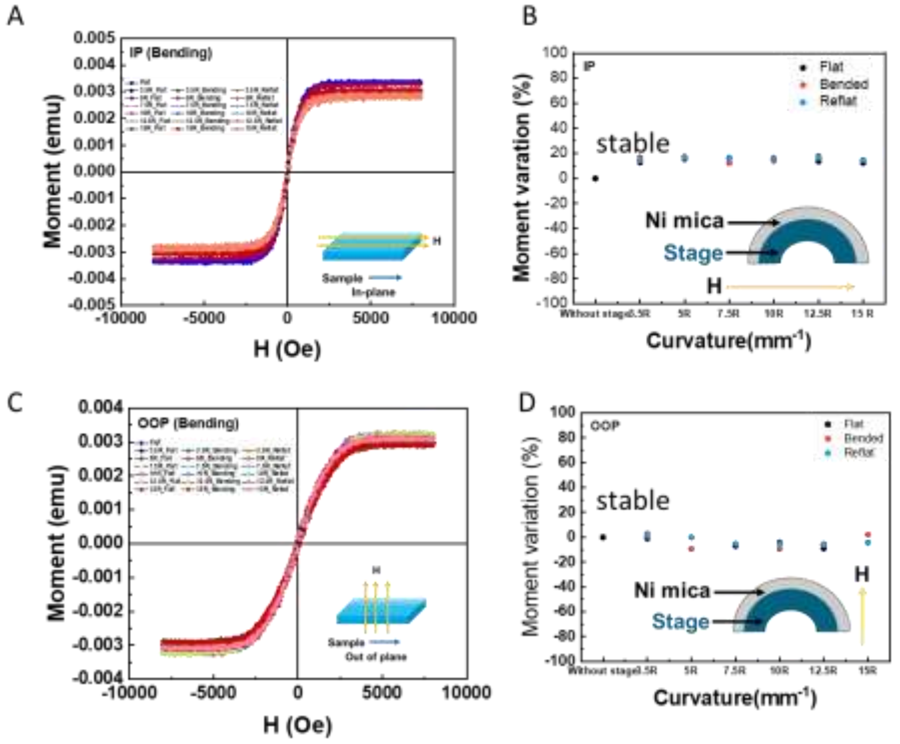

Figure S11. The magnetic properties of metallic Ni intercalated mica:(A) IP hysteresis loops under various bending radii, (B) variation in saturation magnetization under bending along the IP direction, (C) OOP hysteresis loops under various bending radii, (D) variation in saturation magnetization under bending along the OOP direction,

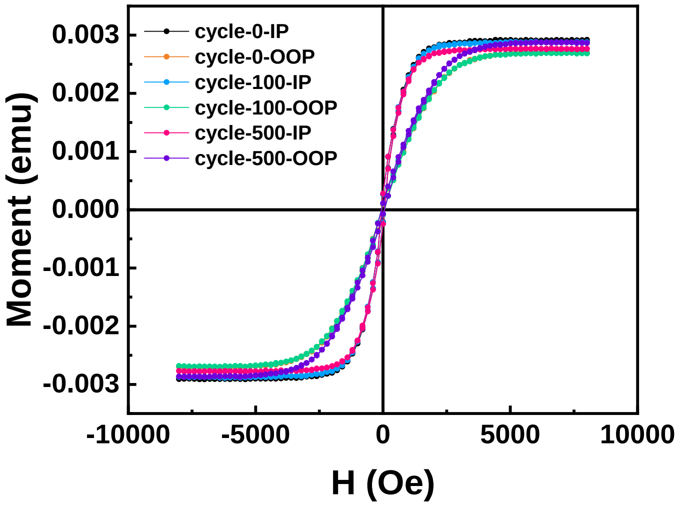

Figure S12. Magnetic cycling bending test.

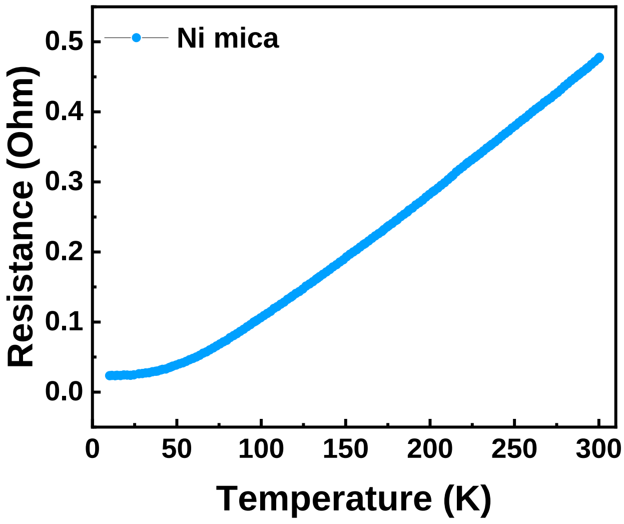

Figure S13. Temperature-dependent longitudinal resistance.

1. Kresse, G.; Furthmüller, J. Efficiency of ab-initio total energy calculations for metals and semiconductors using a plane-wave basis set. *Computational Materials Science* **1996,** 6 (1), 15-50.
2. Kresse, G.; Furthmüller, J. Efficient iterative schemes for ab initio total-energy calculations using a plane-wave basis set. *Physical Review B* **1996,** 54 (16), 11169.
3. Kresse, G.; Hafner, J. Norm-conserving and ultrasoft pseudopotentials for first-row and transition elements. *Journal of Physics: Condensed Matter* **1994,** 6 (40), 8245.
4. Perdew, J. P.; Yue, W. Accurate and simple density functional for the electronic exchange energy: Generalized gradient approximation. *Physical Review B* **1986,** 33 (12), 8800.
5. Tadic, M.; Nikolic, D.; Panjan, M.; Blake, G. R. Magnetic properties of NiO (nickel oxide) nanoparticles: Blocking temperature and Neel temperature. *Journal of Alloys and Compounds* **2015,** 647, 1061-1068 DOI: https://doi.org/10.1016/j.jallcom.2015.06.027.
6. Dubey, P.; Kaurav, N.; Devan, R. S.; Okram, G. S.; Kuo, Y. K. The effect of stoichiometry on the structural, thermal and electronic properties of thermally decomposed nickel oxide. *RSC Advances* **2018,** 8 (11), 5882-5890 DOI: 10.1039/C8RA00157J.
7. Thota, S.; Shim, J. H.; Seehra, M. S. Size-dependent shifts of the Néel temperature and optical band-gap in NiO nanoparticles. *Journal of Applied Physics* **2013,** 114 (21), DOI: 10.1063/1.4838915.
8. Pishko, V. V.; Gnatchenko, S. L.; Tsapenko, V. V.; Kodama, R. H.; Makhlouf, S. A. Temperature dependence of magnetic resonance in NiO nanoparticles. *Journal of Applied Physics* **2003,** 93 (10), 7382-7384 DOI: 10.1063/1.1558253.
9. Klausen, S. N.; Lindgård, P.-A.; Lefmann, K.; Bødker, F.; Mørup, S. Temperature Dependence of the Magnetization of Disc Shaped NiO Nanoparticles. *Physica Status Solidi (a)* **2002,** 189 (3), 1039-1042 DOI: https://doi.org/10.1002/1521-396X(200202)189:3.
10. Feygenson, M.; Kou, A.; Kreno, L. E.; Tiano, A. L.; Patete, J. M.; Zhang, F.; Kim, M. S.; Solovyov, V.; Wong, S. S.; Aronson, M. C. Properties of highly crystalline NiO and Ni nanoparticles prepared by high-temperature oxidation and reduction. *Physical Review B* **2010,** 81 (1), 014420 DOI: 10.1103/PhysRevB.81.014420.
11. Karthik, K.; Selvan, G. K.; Kanagaraj, M.; Arumugam, S.; Jaya, N. V. Particle size effect on the magnetic properties of NiO nanoparticles prepared by a precipitation method. *Journal of Alloys and Compounds* **2011,** 509 (1), 181-184 DOI: https://doi.org/10.1016/j.jallcom.2010.09.033.
12. Yi, J. B.; Ding, J.; Feng, Y. P.; Peng, G. W.; Chow, G. M.; Kawazoe, Y.; Liu, B. H.; Yin, J. H.; Thongmee, S. Size-dependent magnetism and spin-glass behavior of

amorphous NiO bulk, clusters, and nanocrystals: Experiments and first-principles calculations. *Physical Review B* **2007,** 76 (22), 224402 DOI: 10.1103/PhysRevB.76.224402.

13. Rubinstein, M.; Kodama, R. H.; Makhlouf, S. A. Electron spin resonance study of NiO antiferromagnetic nanoparticles. *Journal of Magnetism and Magnetic Materials* **2001,** 234 (2), 289-293 DOI: https://doi.org/10.1016/S0304-8853(01)00313-4.

14. Cooper, J. F. K.; Ionescu, A.; Langford, R. M.; Ziebeck, K. R. A.; Barnes, C. H. W.; Gruar, R.; Tighe, C.; Darr, J. A.; Thanh, N. T. K.; Ouladdiaf, B. Core/shell magnetism in NiO nanoparticles. *Journal of Applied Physics* **2013,** 114 (8), DOI: 10.1063/1.4819807.
